# Post-partum Hamman’s Syndrome

**DOI:** 10.7759/cureus.33144

**Published:** 2022-12-30

**Authors:** Sara Gomes, Tamiris Mogne, Ana Carvalho, Bernardo Pereira, Armindo Ramos

**Affiliations:** 1 Internal Medicine, Centro Hospitalar Barreiro-Montijo, Barreiro, PRT; 2 General Surgery, Unidade Local de Saúde do Norte Alentejano - Hospital Dr. José Maria Grande, Portalegre, PRT; 3 Critical Care, Hospital de Cascais Dr. José de Almeida, Cascais, PRT

**Keywords:** subcutaneous emphysema, labour, pregnancy, spontaneous pneumomediastinum, hamman's syndrome

## Abstract

Hamman's syndrome is an uncommon complication of labor. Its diagnosis is based on clinical suspicion and CT imaging. It is often a benign and self-limiting condition occurring in healthy patients. The risk factors are nulliparity and a prolonged second stage of labor. Hamman's syndrome has life-threatening implications that underscore the importance of early diagnosis and management to avoid any difficulty.

This paper discusses the case of a 21-year-old, healthy female without allergies who was admitted to the hospital in spontaneous labor at 40 weeks of gestation. During the second stage of labor, she developed Hamman's syndrome and presented with subcutaneous emphysema, which led to an emergency C-section. After a chest and cervical CT scan that showed extensive subcutaneous emphysema and a pneumomediastinum, the patient was admitted to the ICU.

## Introduction

Hamman's syndrome is an uncommon complication of labor and delivery that Louis Hamman first described in 1945 [1,2]. It is characterized by pneumomediastinum and subcutaneous emphysema [2]. Its incidence is estimated at one in 100,000 vaginal deliveries [2-4]. It occurs more frequently in nulliparous women with fetal macrosomia in the second stage of labor [1]. The condition seems to be provoked by Valsalva maneuvers, such as vigorous coughing, vomiting, sneezing, physical activity, and vaginal labor [1,2,4]. Despite its presentation, it is usually self-limiting and rarely requires invasive treatment [1,2,4], and a conservative approach is the gold standard.

The mechanism involved is thought to be due to excessive intrathoracic pressure associated with sustained Valsalva maneuvers, leading to marginal alveoli rupture [2,4]. The air dissects through tissue planes into the mediastinum, a phenomenon also known as the Macklin effect.

Subcutaneous emphysema occurs when air escapes from the mediastinum into the subcutaneous and dermal layers of the skin [2,4]. When subcutaneous emphysema in the neck and pneumothorax coexists, both are likely secondary to mediastinal emphysema, with perforation of the mediastinal pleura resulting in pneumothorax [4]. Symptoms include chest pain, dyspnea, cough, palpitations, and palpable crepitus of the face and neck, suggestive of subcutaneous emphysema [4]. Diagnosis is usually made with a complete clinical history, chest x-ray [2,4], and CT scan [1].

## Case presentation

A 21-year-old, healthy primigravida, non-smoker with no known allergies, presented to the hospital in spontaneous labor at 40 weeks of gestation. During the second stage of labor, she presented with sudden retrosternal pain and subsequent subcutaneous emphysema of the chest, neck, and face, leading to an emergency C-section, which occurred without complications.

**Figure 1:**
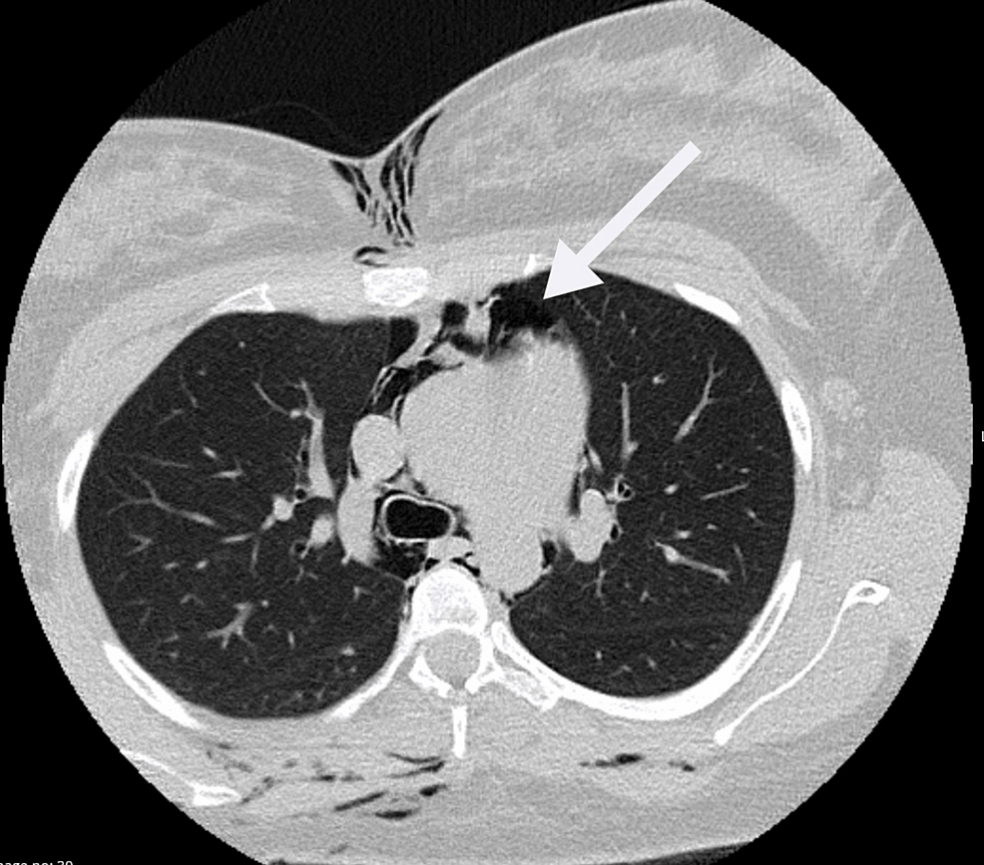
Pneumomediastinum

**Figure 2:**
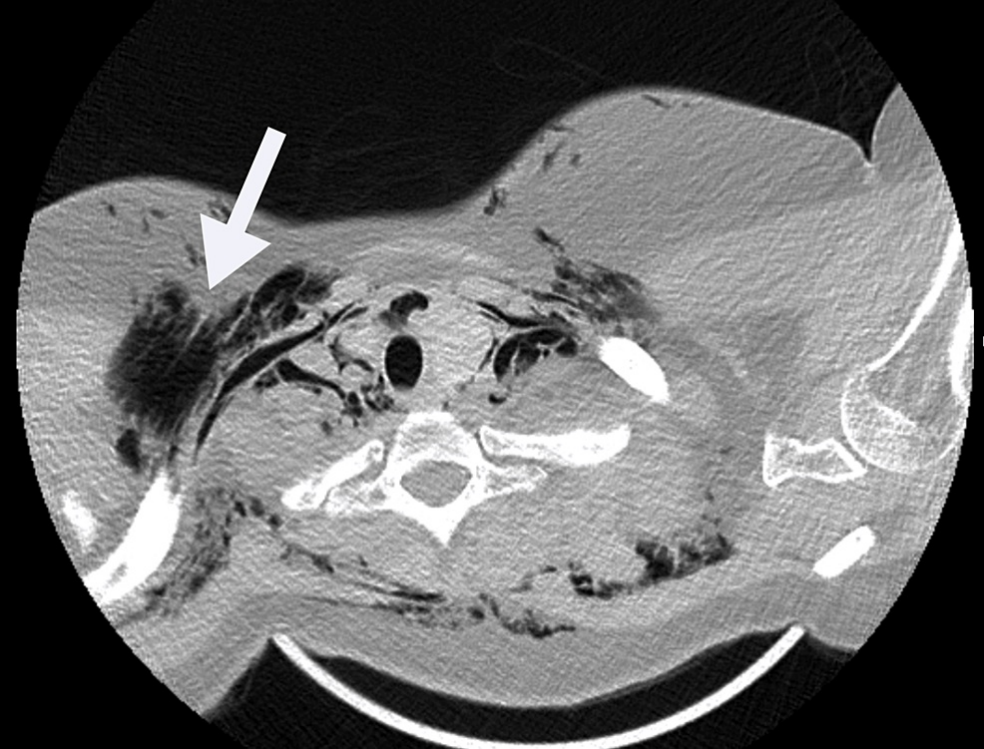
Subcutaneous emphysema

**Figure 3:**
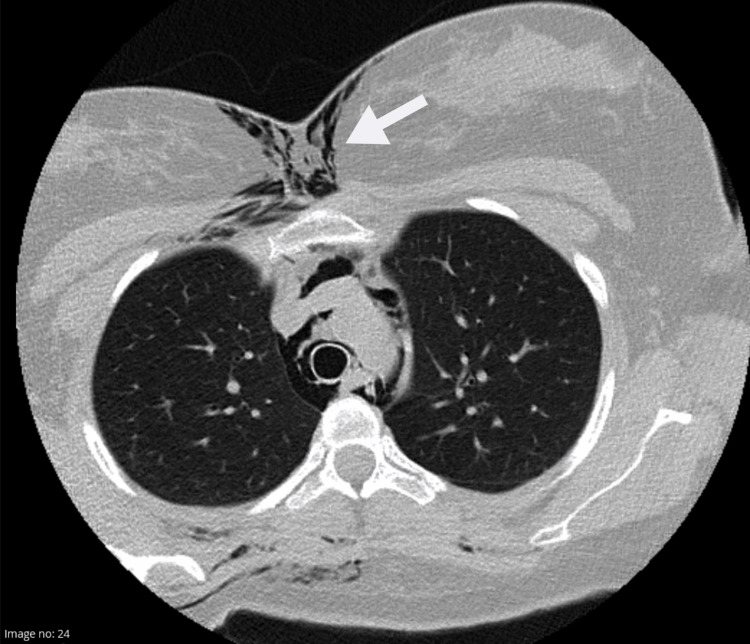
Subcutaneous emphysema

After the C-section, chest and neck CT scans revealed extensive subcutaneous emphysema and a pneumomediastinum (Figures 1-3), resulting in ICU admission. On admission to the ICU, besides exuberant cutaneous emphysema of the chest and face, the patient presented bilateral crackles on the chest auscultation. The remaining presentation was unremarkable for dyspnea, pain, voice changes, cough, tachycardia, or other symptoms. Hamman’s sign, a fine auscultatory crepitation synchronous with the heartbeat [1,4], was also not present. She stayed for observation in the ICU for 24 hours, and oxygen therapy, oral intubation, or other organ support was not required.

## Discussion

Hamman's Syndrome is an uncommon complication of labor and delivery [2]. Its diagnosis is based on clinical suspicion and imaging [4]. Symptoms may include cough, dyspnea, chest/substernal pain, dysphonia, and dysphagia. It is often a benign self-limiting condition and occurs in healthy patients [1]. The risk factors are nulliparity and a prolonged second stage of labor.

The prognosis of this entity is usually benign. However, a differential diagnosis that includes more common conditions is essential [2-4]. The authors highlight the importance of considering the following: abdominal aortic aneurysm dissection, acute myocardial infarction, amniotic fluid embolism, esophageal rupture (Boerhaave's syndrome), mediastinitis, and pneumothorax.

The acute retrosternal pain might have been indicative of acute myocardial infarction or aortic dissection. However, although initially intense, the pain had a rapid resolution and was not the chief complaint in this clinical scenario. Furthermore, no enlargement of the mediastinum was observed on chest radiography, and physical examination was unremarkable for change in pulse on palpation.

Amniotic fluid embolism, also a diagnosis of exclusion, should be suspected in the recent postpartum period in women with respiratory difficulty, which leads to respiratory failure, often culminating in cardiorespiratory arrest and presenting with disseminated vascular coagulation. These classic symptoms may not always be present, and presentation may be just hypotension with acute respiratory failure. In the present case, postpartum dyspnea could lead to this diagnostic hypothesis.

Boerhaave’s syndrome is also a difficult diagnosis, in this case, equated by the presence of retrosternal pain and subcutaneous emphysema. However, the absence of uncontrollable vomiting (the presence of which leads to increased intrathoracic pressure and subsequent pneumomediastinum) reduces the probability of this diagnosis. A Boerhaave's syndrome diagnosis is based on CT imaging that can demonstrate esophageal wall edema and periesophageal fluid, which in the present case excluded it. It should be noted that this syndrome can occur rarely in postpartum with just 200 cases reported globally [5].

Mediastinitis is characterized by an infection in the mediastinum; there could be subcutaneous emphysema, but it is usually associated with local inflammatory signs (edema, erythema, redness of the chest) and fever. Most cases are related to surgical procedures. Spontaneous pneumothorax is more frequent in tall, slender male smokers. The presence of subcutaneous emphysema is not essential; however, retrosternal pain, the Valsalva maneuver, and dyspnea make this diagnosis possible, but here it is again excluded by imaging and physical examination (chest auscultation).

## Conclusions

The treatment of this condition is usually analgesia, supplemental oxygen, and bed rest, with no need for invasive treatment. Complications are rare but can be life-threatening in healthy nulliparous women during their first delivery. A complete differential diagnosis, excluding etiologies with a worse prognosis, and the perception of the existence of this syndrome is an asset for any clinician. Hence, the importance of awareness of this syndrome.
